# Associative-memory deficit as a function of age and stimuli serial position

**DOI:** 10.1371/journal.pone.0268557

**Published:** 2022-08-12

**Authors:** Jonathan Guez, Rotem Saar-Ashkenazy, Yael Poznanski

**Affiliations:** 1 Department of Psychology, Achva Academic College, Beer-Tuvia, Israel; 2 Faculty of Health Sciences, Ben-Gurion University of the Negev, Beer-Sheva Mental Health Center, Beer-Sheva, Israel; 3 Faculty of Social Work, Ashkelon Academic College, Ashkelon, Israel; University of Zurich, SWITZERLAND

## Abstract

Studies have shown associative-memory decline in aging. While the literature is inconclusive regarding the source of the deficit, some researchers argue that it is caused by impaired encoding and maintenance processes in working-memory (WM). Successful retrieval of a stimulus depends on its sequential presentation in the learning list: stimuli at the beginning or the end of the learning list benefit from higher retrieval probability. These effects are known as “primacy” and “recency” effects, respectively. In the case of the primacy-effect, stimuli at early list positions benefit from extensive rehearsal that results in enhanced consolidation and trace in long-term memory (LTM). In the case of the recency-effect, target stimuli at later serial positions are still maintained in WM and can therefore be effortlessly retrieved. Considering these effects could shed light on the involvement of WM in associative-binding. Both behavioral and neuroimaging researchers have studied associative-decline in aging. However, no work has explicitly tested age differences in memory for items versus associations as a function of stimuli serial position (SSP). In the current study, 22 younger and 22 older adults were recruited to participate in a study aimed to test the separate and joint effects of both SSP and aging on memory-recognition of items and associations. In the task used, retrieval was manipulated for SSP (beginning/middle/end of the list) and item/associations recognition modes. We hypothesized that greater associative-decline will be observed in older adults, specifically for recently presented material. The results showed that both groups presented a significant associative-deficit at the recency positions; this decrease was additive and did not correspond to the expected interaction effect. Further analysis showed that the source of associative-memory decline for stimuli at recency position in older adults resulted from an increase in false-alarm (FA) rates. These results support the involvement of WM-binding impairment in aging.

## Introduction

Numerous studies have shown age-dependent episodic memory decline [1]. Several hypotheses have been suggested to explain older adults’ poor memory performance: reduction in attentional resources or processing abilities [2, 3], reduction in processing speed [4] and failure of inhibitory processes [5]. Notwithstanding, not all memory processes and components are similarly affected by age [6, 7], rather greater memory decline is observed in older adults when trying to generate and retrieve links between units of information as opposed to the retrieval of single units of information [8–11]. Multifaceted/complex episodes are based on multiple kinds of (eventually related) information sources. Remembering an episode requires remembering the individual pieces of information (i.e., “items”) as well as their associations with each other and other specific or contextual information (i.e., “associations”) [12, 13]. Studies conducted with different age groups have shown that older adults recalled significantly fewer targets and links between targets than did younger adults [14–16]. These empirical research findings in older adults brought Naveh-Benjamin [17] to conclude that with age, older adults mainly fail to encode and retrieve links between units of information (i.e., associative-memory). The associative-decline was formulated as the difference between younger and older adults in memory recognition for single units of information (i.e., items) versus associations between stimuli and was named the *Associative-Deficit Hypothesis* (ADH). This age-related associative-deficit is extensively supported by behavioral data [18–23] and applies to memory for paired-stimuli as well as to memory for source, context, temporal order and location, all of which require binding processes [10].

The literature is inconclusive regarding the source of the disproportionate deficit in associative versus single (i.e., items) units of information recognition. Some researchers argue that it is caused by impaired encoding and maintenance processes in working memory (WM). Cowan, Naveh-Benjamin, Kilb, & Saults [24] used visual objects and their spatial location in WM to assess for item change and binding deficits in younger and older adult participants. The results of the experiment showed a strong bias toward the detection of changes, especially binding changes. Chen and Naveh-Benjamin [25] used a continuous recognition paradigm and replicated an associative WM decline in three experiments. Brockmole, Parra, Della Sala, & Logie [26] used a change detection task to assess associative-memory decline and reported that older adults store proportionally fewer bound representations than individual features compared to younger adults. Lastly, Hara & Naveh-Benjamin [27] simulated long-term memory (LTM)- dependent associative-memory deficit (as seen in older adults) in young adults by manipulating WM resources, thus highlighting the involvement of WM in successful associative recognition. In contrast to these findings, other researchers did not find any associative-deficit in WM, which conflicts with the WM explanation. Bopp & Verhaeghen [28] addressed the WM associative-deficit question using a repetition-detection task that can differentiate memory for content from memory for context. In three experiments the authors did not find a specific age-related deficit for context in WM. Parra, Abrahams, Logie & Sala [29] addressed visual short-term memory using color-shape conjunctions to test associative-binding deficits in WM and also concluded that binding in WM is not age-dependent.

Taking into account the original serial position curve effects could shed light on the involvement (or lack of involvement) of WM in associative-binding. It is well known that the successful retrieval of a stimulus depends on its sequential presentation (beginning/middle/end of the list) in the learning list [30, 31]. Stimuli at the beginning [32, 33] or the end [30, 34–36] of the learning list benefit from higher retrieval probability compared to stimuli presented at intermediate positions. These effects are known as “primacy” and “recency” effects respectively. Neuroimaging studies have documented the involvement of the prefrontal cortex (PFC) [37–40] and structures in the medial temporal lobe (MTL), with a specific contribution of the hippocampus [41–44]. In the case of the primacy effect, extensive rehearsal for stimuli at early list positions results in better retrieval probability; thus, the primacy explanation links LTM formation and WM processes [45–47]. Neuroimaging studies applying functional magnetic resonance imaging (fMRI) support differential involvement of MTL structures in this process by showing that WM maintenance facilitates the encoding of a stimulus into LTM by activating rehearsal processes in the hippocampus (for associations between stimuli [48]) or in the parahippocampal cortex (for single units of information [49]). In the case of the recency effect, target stimuli at late serial positions (i.e., at the end of the learning list) also benefit from higher retrieval probability because they are still maintained in WM and therefore can be effortlessly retrieved, compared to stimuli at intermediate positions [30, 46, 50–52].

Reports of studies that tested the effect of the serial position of a stimulus on memory in older adult participants have indicated that while the primacy effect was absent, the recency effect remained intact [53, 54]; these studies however did not account for associative-memory. Lately, we simulated an associative-memory deficit (as seen in older adults) in young adult participants by controlling stimuli serial position (SSP) and presentation duration. This resulted in greater memory decline for associative material presented at the end of the learning list compared to single-unit targets (i.e., items) at similar positions [55]. These results highlight the different benefits single units of information have from a late sequential position during learning as compared to paired units of information. The results raise a question regarding the nature of both intact as well as impaired associative-binding in WM.

Because the recency effect reflects retrieval from WM, it is expected that impaired WM will contribute to the associative-deficit in older age; in that case, the age-related associative-deficit should be greatest for the recency portion of the list compared to the beginning and middle portions of the list. If the age-related associative-deficit is similar in all parts of the list, regardless of the serial position of the studied information, then this deficit cannot be attributed solely to impaired WM. Whereas both behavioral and neuroimaging researchers have studied associative-decline in aging, currently no work has explicitly compared age differences in memory for items and associations as a function of SSP. In the current study, 22 younger and 22 older adults were recruited to participate in a study aimed to test the separate and joint effect of both SSP and aging on memory recognition for items versus associations. We hypothesized that greater associative-decline (compared to the expected decline in memory for items with similar serial location) will be observed in older adults, specifically for recently presented material (i.e., stimuli that was presented at the end of the learning list).

## Materials and methods

### Participants

The number of participants was calculated based on our last study which was conducted with young adult participants and had tested similar effects [55]. Calculation of the estimated effect and sample size was conducted using MedCalc software and considered both type-1 (α. 0.05) and type-2 (β, 0.1) errors as well as the estimated difference between means (0.26, based on previous research) and corresponding SD (0.18, 0.29 based on previous research) for each group. Using G*power software we calculated the estimated minimum number of participants for the total sample according to the estimated effect size (η^2^_*p*_ = 0.25) of the highest hypothesized interaction (F-test, ANOVA: repeated measures, within-between interaction). This calculation resulted in ~20 participants for each group and served as the basis for the estimated number of participants that were invited to take part in the current study. Based on this calculation, 46 participants were invited to participate in the current study, of which 22 were younger (M_(years)_ = 24.90±2.12 SD, 9 women) and 24 were older (M_(years)_ = 73.61±8.10 SD, 14 women) adults. All reports were given by the participants via self-report. All participants reported normal and intact everyday functioning with no disabilities and/or psychiatric disorders. Participants in the younger adult group were Achva Academic College students that were rewarded for their participation with course credit, an acceptable procedure in a first-year introductory psychology academic course. The older adults group consisted of participants recruited from the local community and senior citizens’ home. Participants and their caregivers were asked about everyday functioning. They were asked to describe and report any cognitive/physical disabilities. Exclusion criteria included past/current psychiatric or neurological disorders, current sensory/motor disorders and/or a formal diagnosis of learning disabilities. Participants that met one or more exclusion criteria in their description were excluded from the study. Two (older) participants were excluded from the study and were not included in the analysis after their caregivers reported that they were diagnosed with mild cognitive impairment (MCI). Finally, 22 participants for each group were included in the analysis. The study was approved by the local institutional review board of Achva Academic College. All participants gave their written informed consent for participation in the study.

### Experimental design and hypotheses

Three independent variables were used in this study: *SSP* (beginning, middle and end of the learning list; a within-subject variable); X *test* (item versus associative recognition; a within-subject variable); and X *age* (younger versus older adults; a between-subject variable). The dependent variable was memory accuracy, calculated as “hit minus false-alarm (FA)” rate for each participant in each experimental condition. In addition, and to specifically address a cumulative associative-decline, we computed *associative-deficit index* (ADI) reflecting the difference between item recognition and associative recognition performance. The ADI was calculated by subtracting the proportion of Hits minus the proportion of false alarms in associative recognition trails, from the proportion of Hits minus the proportion of false alarms in item recognition trails (proportion of Hits-FA_*item*_ minus proportion of Hits-FA_*association*_). Higher ADI scores reflect greater associative-deficit. While differences in item and associative recognition were evident for all participants regardless of age, our main hypothesis was that the greatest difference between item and associative recognition (i.e., associative-deficit, as measured via ADI scores) would be evident for older adults and for stimuli located at the end of the learning list (compared to material located at the beginning/middle of the learning list).

### Memory task

**Fig 1:**
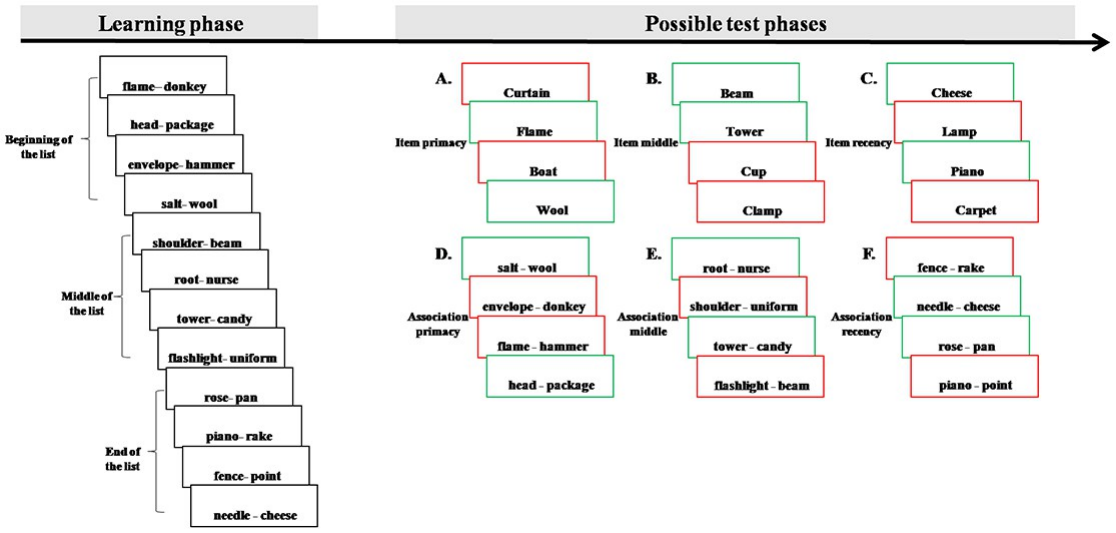
A description of the experimental paradigm. 24 separate lists of 12 pairs of words were used. Each list was then immediately followed by only one of six (A-F) tests options (3_(stimuli serial position)_ X 2_(test)_). The four replications for each of the six conditions were presented randomly across participants and each stimulus was used only for one of the tests (i.e., each stimulus was only used once in the experiment). Green rectangles represent targets while red rectangles represent distracters.

24 separate lists of 12 pairs of words were built from a pool that contained 576 words that were unrelated visually, semantically and auditorily. The lists consisted of high-frequency Hebrew common nouns (based on the Hebrew norms [56]). After each study list, participants immediately performed an item or an associative test for stimuli located in differential list positions; beginning (4 first pairs of the learning list, to assess the primacy effect), middle (4 middle pairs of the learning list) and end (4 last pairs of the learning list, to assess the recency effect) of the learning list. Stimuli were presented visually and displayed centrally on a 15” computer screen one at a time. The four replications for each of the six conditions (3_(stimuli serial position)_ X 2_(test)_) were presented randomly across participants and each stimulus was used only for one of the tests (i.e., each stimulus was only used once in the experiment). Fig 1 describes the experimental paradigm.

### Item recognition test

Participants were presented with 4 words taken from only one list position (beginning/middle/end). Of the 4 words, 2 words were targets, i.e., words that had appeared in the learning list, and 2 were distracters, i.e., newly introduced words. Participants were informed that the list include targets and distracters, and were instructed to respond as quickly and accurately as possible to each stimulus with a designated “yes” key for targets and a “no” response key for distracters.

### Associative recognition test

Participants were presented with 4 pairs of words taken from only one list position (beginning/middle/end). Of the pairs, 2 pairs were targets, i.e., word pairs that had appeared in the learning list and 2 were distracters, i.e. rearranged pairs. Distracters were items that had appeared in the learning list but were now recombined into novel (distracter) pairs. Recombined pairs were created from similar list positions and were not mixed across the list. Participants were again informed that the list included targets and novel pairs, and were instructed to respond as quickly and accurately as possible to each pair with the same keys as in the item recognition test.

### Procedure

Younger participants were tested individually in designated rooms at Achva Academic College and older participants in the senior citizens’ home. Participants were given instructions and also clarifications as needed before the start of the first learning list. Participants were informed that they were about to view 24 learning lists and that each list would be followed by either an item or an association test. Participants were asked to learn and remember both the individual items as well as the pairs presented in the learning list for the upcoming tests. In each test, participants viewed 12 word pairs on the computer monitor, one at a time, at a rate of 2 seconds per pair. All tests in the current experiment were self-paced; that is, the tested material appeared on the screen for 1.5 seconds. A response during this time range (1.5 seconds) caused the appearance of the next stimulus. If no response was recorded within 1.5 seconds, the stimulus disappeared from the screen, and the next stimulus appeared only after a response had been recorded. In all test steps, only the response to a stimulus caused the appearance of the next one. In each memory test type (item recognition or associative recognition) participants were tested for only one of the three stimuli locations: four words located at the beginning of the learning list (for testing the primacy effect), four words located at the middle of the learning list and an additional four words located at the end of the learning list (for testing the recency effect). Each stimuli list location was tested individually with a different test. Participants were blind to stimulus location before the actual test phase, i.e., they were presented with an item or an association test, but were not introduced to the stimulus location from the learning list until the beginning of the test.

### Statistical analysis

All data were analyzed using STATISTICA software (version 12), an advanced analytics software package originally developed by StatSoft and currently maintained by TIBCO Software Inc. Between-group comparisons (young versus older adults) were conducted with Students’ T-test and the hypothesized interactions between the independent variables that were used in this study were calculated with F-tests.

## Results

Table 1 shows the demographic characteristics for the 22 young and 22 older adult participants that were included in the analysis (including statistical analysis). The groups significantly differed in age but did not differ in years of education and no differences in gender distribution were found between groups. In order to assess memory accuracy, we computed the “hit minus false-alarm” rate for each participant in each experimental condition. A hit occurs when the participant correctly identifies a target test item as a target and a false-alarm (FA) occurs when a distracter is erroneously identified by the participant as a target. With this measure, chance level performance (guessing) yields a score of 0.00 and perfect performance yields a score of 1.00. This equated the item and the associative recognition tests with respect to the scale used. The ADI was calculated as the difference between item and associative recognition (item recognition performance minus associative recognition performance).

**Table 1:** Demographic characteristics of participants.

*Demographical characteristics*	Young Adults (n = 22)	Older Adults (n = 22)	p-value
Age (years) ^a^	24.9 [2.12]	72.0 [8.14]	<.001
Education (years) ^a^	13.43 [1.37]	12.41 [3.48]	.19
Gender ^b^	9 women [41%]	14 women [64%]	.13

*Note*. Table 1 shows demographic characteristics for young and older adult participants.

^a^ = variables are depicted by Mean±SD and compared via Students’ T-test;

^b^ = variables are depicted by n(%) and compared via Pearson’s Chi-square;

**Fig 2:**
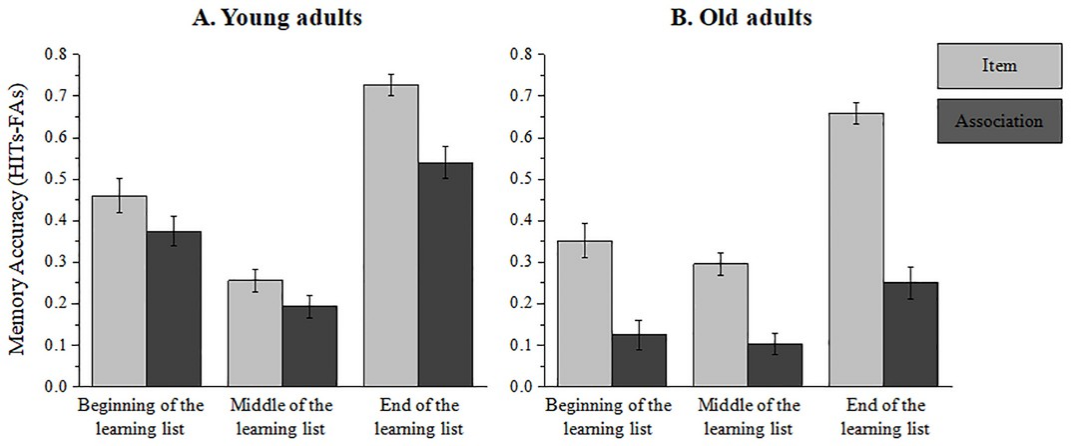
Memory accuracy as a function of *age* (a = young, b = old), *SSP* (beginning, middle and end of the learning list) and *test* (item/association). Error bars represent the standard error of the mean.

To specifically address the role of *SSP* (beginning/middle/end of the learning list) on memory recognition for items versus associations in younger and older adult participants, we computed a three-way mix design ANOVA with the above factors. Reaction-time (RT) means and standard deviations for item and associative recognition as a function of age and SSP are available in Table 2. The results of the three-way mix design ANOVA (see Table 3 and Fig 2) indicated three main effects. A significant main effect for *age* [*F*(1, 42) = 39.93, *p*<.001, η^2^_*p*_ = .49], with lower overall memory performance in the older group [M = 0.29±0.05SD] compared to the younger group [M = 0.42±0.07SD]. A significant main effect for *test* [*F*(1, 42) = 136.50, *p*<.001, η^2^_*p*_ = .76], with higher memory recognition for items [M = 0.45±0.09SD] than for associations [M = 0.26±0.14SD]. A significant main effect for *SSP* [*F* (2, 84) = 93.33, *p*<.001, η^2^_*p*_ = .68], with planned-comparison analysis showing that memory accuracy for stimuli located at the end of the learning list [M = 0.54±0.14SD] was higher than for stimuli located at the beginning of the learning list [M = 0.32±0.16SD] and middle of the learning list [M = 0.21±0.08SD] [*F*(1, 42) = 147.31, *p*<.001, η^2^_*p*_ = .77]. In addition, memory for stimuli located at the beginning of the learning list was significantly higher than for stimuli located at the middle of the learning list [*F*(1, 42) = 25.17, *p*<.001, η^2^_*p*_ = .37].

**Table 2:** Reaction-time (RT) means and standard deviations for item and associative recognition as a function of age and SSP.

		*M*	*(SD)*	*Minimum*	*Maximum*
***Young adults***					
Beginning of the list	Item	1115.81	222.25	784	1654
	Association	1458.18	269.61	959	1960
Middle of the list	Item	1125.27	234.30	767	1612
	Association	1508.68	269.38	978	1956
End of the list	Item	987.40	167.74	753	1442
	Association	1387.77	370.34	887	2432
***Old Adults***					
Beginning of the list	Item	1604.68	355.27	1033	2432
	Association	2002.90	395.47	1239	2653
Middle of the list	Item	1608.31	393.67	978	2357
	Association	2016.31	351.20	1340	2766
End of the list	Item	1490.86	322.55	971	2085
	Association	1987.27	376.73	1435	2876

**Table 3:** Means and standard deviations for Hits and False Alarms (FA) for item and associative recognition as a function of age and SSP.

		Beginning of the learning list		Middle of the learning list		End of the learning list	
		*M*	*(SD)*	*M*	*(SD)*	*M*	*(SD)*
***Young adults***							
Item Memory	Hits	.86	(.23)	.72	(.29)	.96	(.05)
	FA	.40	(.24)	.46	(.30)	.23	(.16)
	Hits-FA	.46	(.24)	.25	(.14)	.73	(.14)
Associative-memory	Hits	.66	(.19)	.68	(.26)	.80	(.25)
	FA	.28	(.21)	.48	(.28)	.26	(.18)
	Hits-FA	.38	(.19)	.19	(.13)	.54	(.19)
***Old Adults***							
Item-memory	Hits	.74	(.26)	.64	(.27)	.93	(.10)
	FA	.39	(.21)	.35	(.25)	.28	(.13)
	Hits-FA	.35	(.12)	.29	(.10)	.65	(.10)
Associative-memory	Hits	.61	(.33)	.68	(.27)	.75	(.28)
	FA	.49	(.30)	.58	(.25)	.50	(.33)
	Hits-FA	.12	(.12)	.10	(.11)	.25	(.16)

FA = False Alarms.

We observed a significant two-way interaction between *SSP* and *age* [*F*(2, 84) = 6.43, *p*<.001, η^2^_*p*_ = .13], showing a robust memory performance difference between age groups (younger adults>older adults) for stimuli located at the beginning [*F*(1, 42) = 17.21, *p*<.001, η^2^_*p*_ = .29] and end of the learning list [*F*(1, 42) = 26.50, *p*<.001, η^2^_*p*_ = .38] but not for stimuli located at intermediate positions [*F*(1, 42) = 1.09, *p* = .30, η^2^_*p*_ = .02]. The second two-way significant interaction was observed between *test* and *age* [*F*(1, 42) = 24.58, *p*<.001, η^2^_*p*_ = .37]. Planned-comparison analysis showed that older and younger adult participants did not significantly differ in memory recognition for items [*F*(1, 42) = 3.33, *p* = .07, η^2^_*p*_ = .13] while a sharp group difference in performance was evident for associative recognition [*F*(1, 42) = 58.84, *p*<.001, η^2^_*p*_ = .58]. The third significant two-way interaction was found between *test* and *SSP* [*F*(2, 84) = 8.40, *p*<.001, η^2^_*p*_ = .16]. Planned-comparison analysis showed a significant difference between item and associative recognition at early list positions [*F*(1, 42) = 23.27, *p*<.001, η^2^_*p*_ = .35], middle list positions [*F*(1, 42) = 20.35, *p*<.001, η^2^_*p*_ = .32], and later/end list positions [*F*(1, 42) = 92.42, *p*<.001, η^2^_*p*_ = .68]. Despite significant differences in all three comparisons, the effect size of the later end of the list positions was the largest (.68, compared to the two other conditions: early; .35 and middle; .32 list positions). This pattern replicates our previous findings [55] and enables us to generalize the reported results of greater associative-decline for stimuli located at the end of the learning list to older adult participants.

Although the two-way interaction between *test* and *age* was significant under all serial positions (primacy, F(1, 42) = 4.81, p = .033, η^2^_*p*_ = .10; middle, F(1, 42) = 5.31, p = .026, η^2^_*p*_ = .11; and recency, F(1, 42) = 12.75, p = .000, η^2^_*p*_ = .23), the differences were additive and the three-way interaction was not significant [F<1]. To specifically assess our hypothesis regarding greater ADI scores under the recency portion in older adults, we also computed a two-way mixed design ANOVA with *SSP* (beginning/middle/end of the learning list, a within-subjects factor) X *age* (young/old, a between-subjects factor) as independent variables and ADI as the dependent variable. This interaction was not significant [F<1]. Together, these statistical analyses emphasize that the associative-deficit is significantly augmented under the recency portion, but with a similar outcome for both young and old adults.

**Fig 3:**
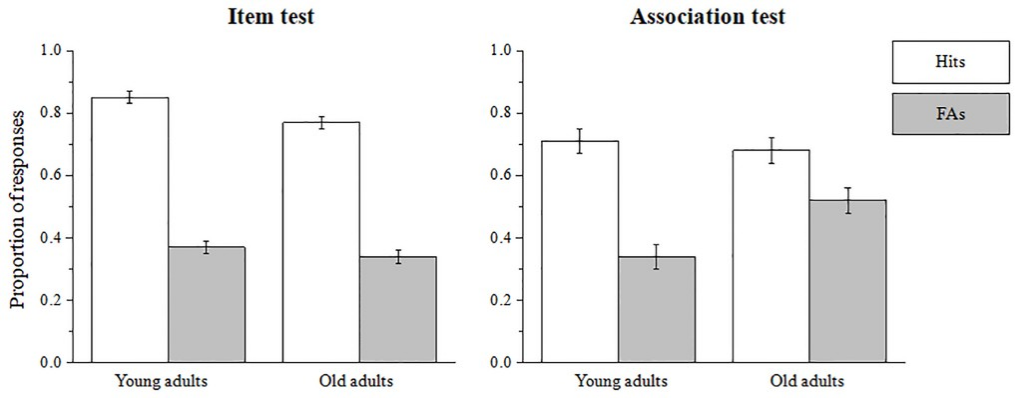
Hit and FA rates as a function of age (young/old adults) and test (item/association). Error bars represent the standard error of the mean.

It is worth noting that higher ADI scores can be evident due to a decrease in Hits rates or increase in FAs responses or both. To assess the locus of the associative-deficit we computed a four-way mix design ANOVA with the following factors: *test* (item versus associative recognition; a within-subject variable), X *age* (younger versus older adults; a between-subject variable), X *SSP* (beginning/middle/end of list, a within-subject variable) and X *response* (Hits versus FAs; a within-subject variable). The four-way interaction did not reach significance (F<1). The results reported here are for the highest interactions that include the *test* factor and that reached significance; the three-way interaction between *test* X *age* X *response* [*F*(1, 42) = 24.57, *p*<.001, η^2^_*p*_ = .37]. Planned-comparison analysis on this interaction showed that while the two-way simple interaction between *age* and *response* in the item condition was not significant [*F*(1, 42) = 3.33, *p* = .07, η^2^_*p*_ = .07], the two-way simple interaction between the same factors was significant under the associative condition [*F*(1, 42) = 58.84, *p*<.001, η^2^_*p*_ = .58]. Further analysis on the later interaction revealed that while no difference between younger and older adults was evident for Hits responses [*F < 1*], significantly higher FA rates were evident for older adult participants under the associative recognition condition [*F*(1, 42) = 10.30, *p*<.001, η^2^_*p*_ = .19], see Fig 3. Another three-way interaction that was found to be significant was between *test* X *SSP* and X *response* [*F*(2, 84) = 8.40, *p*<.001, η^2^_*p*_ = .17]. Planned-comparison analysis showed that the well-established interaction between test (item versus association) and response (Hits versus FA) was most robust for the recency position [*F*(1, 42) = 92.42, *p*<.001, η^2^_*p*_ = .68] compared to primacy and middle positions [*F*(1, 42) = 23.25, *p*<.001, η^2^_*p*_ = .35; *F*(1, 42) = 20.35, *p*<.001, η^2^_*p*_ = .32; respectively].

## Discussion

The objective of the current study was to test the separate and joint effect of both SSP and aging on memory recognition for items versus associations. The results of the current experiment replicate our previous results that showed greater associative memory decline (measured as the ADI score; i.e., reflecting the difference between item and associative recognition performance, regardless of age) for stimuli presented at the end of the learning list [55] and extend them to older adult participants. The results of the previous and the current study together provide behavioral support for the existence of an associative-binding deficit which is predominantly evident for stimuli with later list positions (i.e., recency positions). However, we did not find evidence for an augmented associative-deficit at recency positions in older age which would have supported our hypothesis. Furthermore, in a planned analysis conducted on the Hit versus FA rates, we were able to focus the locus of the associative-deficit in older adults to significantly increased FA rates under the associative condition. These results support the conclusion that generally, older adults were more likely to respond that a recombined pair (namely, a distracter) had appeared in the learning list, especially if the tested material was located at the end of the learning list.

The dual-process theory asserts that single and paired (i.e., associated) units of information are differently treated in memory [57]. The theory claims that two independent contributing processes underlying memory: familiarity and recollection. Familiarity is the recognition of information in the absence of any specific details. It is considered relatively automatic in nature and is often defined as a sense of “being familiar” with the stimuli to be recognized. Recollection refers to the memory of events that is accompanied by specific details and associations. It is considered to involve executive functioning and is associated with an obvious sense of remembering. The difference between these processes is often tested behaviorally by using item-association memory paradigms, suggesting that while item recognition is linked to both familiarity and recollection processes, associative recognition relies solely on recollection [10].

Our results are in line with the dual-process theory [57] and the view that while familiarity is sufficient for the correct recognition of items, correct recognition of associations requires recollection. To correctly recollect associative information, it is not enough to be familiar with its components (i.e., items), rather enough details must be retained to determine whether those items were presented together during learning. Aging appears to disrupt recollection, but not familiarity processes, hence, we attribute older adults’ tendency to mistakenly judge recombined pairs as having appeared in the learning list, to the deterioration in cognitive abilities occurring in older age. Consistent with previous literature [10] we postulate that with age, learning becomes more of a familiarity-based process rather than a recollection-based process, because relying on familiarity is not enough for correct recognition of associations. This claim is exhibited by the pronounced associative (i.e., recollection based) recency deficit found in the current study.

While most studies have investigated the serial position effect using a free recall paradigm, the current study benefits from three exceptional advantages of using a recognition-based memory paradigm. Firstly, it deals with the lack of experimental control in the free recall paradigm (in the free recall paradigm the investigator cannot control the retrieving order of the subject). Secondly, using a recognition-based memory paradigm allowes the ability to compare the associative performance to the single items that form it. That is, the ADI score enables the comparison between item and associative recognition, while in the free recall task this comparison is not possible. Thirdly, stimuli at the test phase were not presented randomly on the screen but rather according to their specific location in the learning list (beginning/middle/end of the list). This procedure enabled us to control primacy and recency effects. Each learning list was followed by only one short test according to the 6 experimental conditions (3X2); although this controlled design is not parsimonious (as it requires a large number of tests) it does, however, decrease the sequential effect (i.e., the effect of the previous stimulus on the upcoming response for the current stimulus) and the preparation of the participant to a specific test.

Studies of the serial position curve assert that in the case of the recency effect, stimuli at late serial positions benefit from higher retrieval probability, since they are still “stored” in WM and thus can be effortlessly retrieved [30, 46, 50–52]. In the current study we were interested in the involvement of WM in the (specific) associative-decline in older age. We tested the separate and joint impact of SSP and aging on memory for single versus paired units of information. Our results show that the recency effect does not fully explain associative memory recognition performance for material presented at later list positions. Despite the overall benefit of information located at the end of a learning list (compared to the rest of the list), for both younger and older adults the associative-deficit was largest for end of list information. The results of the current study clearly show that single and paired-stimuli (i.e., associative material) cannot benefit the same from higher retrieval probability due to their presence in WM. Recognition for associative material decreased dramatically for stimuli from later list positions (compared to memory recognition for single items at similar positions). These findings raise the possibility of the existence of binding deficits already at WM phases as bound stimuli are still maintained in WM during immediate recognition testing.

Evidence regarding an age-related associative-memory deficit in short-term/WM could reveal the locus of the deficit, as the existence of the deficit already in short-term/WM phases, would support an age-related variance in associative encoding abilities. However, the absence of a deficit in short-term/WM would support a long-term age-related associative-deficit that could be a result of age-related differences in consolidation and forgetting rates of associations over time [25]. Our results do not fall under one of these options solely. Our findings do point to a larger associative-deficit for associative material presented at later list positions (i.e., stimuli that are still maintained in WM and lack a trace in LTM), but additively for both young and old participants. These results support general variance in associative encoding abilities, independent of age.

Several limitations of the current study must be acknowledged. Firstly, in the current study no formal-objective cognitive assessment was used, thus we cannot rule out the confounding of an undiagnosed MCI as a contributor to the memory deficits shown in the older adults group. Further studies are encouraged to use a formal-objective cognitive assessment to ensure the general cognition of the participants and to avoid potential confounds rising from general cognitive decline. Secondly, although the current study provides behavioral support to well known aging and memory processes that are largely considered as relying on the brain structures mentioned it did not include imaging. Further neuroimaging studies are warranted to test the presence of associative-binding deficits in both young and old adults and to provide empirical neuro-anatomical and functional connectivity evidence to support or contradict the behavioral findings at brain level. Lastly, in the current study we relied on the widely accepted historical view that supports the division and interpretation of the serial position curve as beginning/middle of list stimuli as corresponding to LTM and end of list stimuli as corresponding to short-term/WM [30–36, 58]. Notwithstanding, other views also exists [59–62], thus our interpretation of the results must be taken with caution.

Whereas the study of aging has traditionally been carried out by searching for specific brain regions that are susceptible to neural degeneration or decrease in brain volume, it is crucial to study their interactions with diverse cognitive paradigms, allowing to identify not only the presence of a deficit, but also its locus and core characteristics. Deep behavioral investigation of the core-features of associative-binding can shed light on the precise nature of, for example, encoding and retrieval deficits, and thus set the ground for neuroimaging studies to test the reported deficits at brain level. As seen in the current study, we were able to emphasize the locus of associative-memory demands to increased FA rates already at WM stages. Since cognitive dysfunctions are a hallmark of older age, such understanding can serve as the basis for the detection of potential biomarkers for diagnosis and prognosis of aging individuals, as well as the development of standard behavioral and functional-imaging protocols and tools aimed to adequately assess these (lacking) abilities in older age.

## Supporting information

S1 Data The data file used for the current study is available in xls. format. (XLS)

S2 Data A meta-data file with information regarding each data-column, its valid inputs and their metric. (PDF)
