# Supplementary material for: Associative-memory deficit as a function of age and stimuli serial position
Source: PLoS One. 2022 Aug 12;17(8):e0268557. doi: 10.1371/journal.pone.0268557 (PMC9374252; doi:10.1371/journal.pone.0268557)
Supplement: S2 Data — (PDF) [file pone.0268557.s002.pdf]

Meta-data file

VARIABLE	METRIC	RANGE (Valid Output)
AGE	age years	Numeric continuous (Range 21-88)
group	Y=Young adults; O=Old adults	TEXT categorical
sex	M=Male; F=Female	TEXT categorical
education	Years of education	Numeric continuous
assoc prime hit	Primacy-Association-HIT (absolute mean of response [4 lists])	Numeric (Range 0-2)
assoc prime FA	Primacy-Association-FA (absolute mean of response [4 lists])	Numeric (Range 0-2)
assoc mid hit	Middle-Association-HIT (absolute mean of response [4 lists])	Numeric (Range 0-2)
assoc mid FA	Middle-Association-FA (absolute mean of response [4 lists])	Numeric (Range 0-2)
assoc rec hit	Recency-Association-HIT (absolute mean of response [4 lists])	Numeric (Range 0-2)
assoc rec FA	Recency-Association-FA (absolute mean of response [4 lists])	Numeric (Range 0-2)
Item prime hit	Primacy-ITEM-HIT (absolute mean of response [4 lists])	Numeric (Range 0-2)
Item prime FA	Primacy-ITEM-FA (absolute mean of response [4 lists])	Numeric (Range 0-2)
Item mid hit	Middle-ITEM-HIT (absolute mean of response [4 lists])	Numeric (Range 0-2)
Item mid FA	Middle-ITEM-FA (absolute mean of response [4 lists])	Numeric (Range 0-2)
Item rec hit	Recency-ITEM-HIT (absolute mean of response [4 lists])	Numeric (Range 0-2)
Item rec FA	Recency-ITEM-FA (absolute mean of response [4 lists])	Numeric (Range 0-2)
%assoc prime hit	Primacy-Association-HIT (Proportion)	Numeric (Range 0-1)
%assoc prime FA	Primacy-Association-FA (Proportion)	Numeric (Range 0-1)
%assoc mid hit	Middle-Association-HIT (Proportion)	Numeric (Range 0-1)
%assoc mid FA	Middle-Association-FA (Proportion)	Numeric (Range 0-1)
%assoc rec hit	Recency-Association-HIT (Proportion)	Numeric (Range 0-1)
%assoc rec FA	Recency-Association-FA (Proportion)	Numeric (Range 0-1)
%Item prime hit	Primacy-ITEM-HIT (Proportion)	Numeric (Range 0-1)
%Item prime FA	Primacy-ITEM-FA (Proportion)	Numeric (Range 0-1)
%Item mid hit	Middle-ITEM-HIT (Proportion)	Numeric (Range 0-1)
%Item mid FA	Middle-ITEM-FA (Proportion)	Numeric (Range 0-1)
%Item rec hit	Recency-ITEM-HIT (Proportion)	Numeric (Range 0-1)
%Item rec FA	Recency-ITEM-FA (Proportion)	Numeric (Range 0-1)
ITEM prime	Proportion HIT minus Proportion FA	Numeric (Range -1- +1)
ASSOC prime	Proportion HIT minus Proportion FA	Numeric (Range -1- +1)
ITEM mid	Proportion HIT minus Proportion FA	Numeric (Range -1- +1)
ASSOC mid	Proportion HIT minus Proportion FA	Numeric (Range -1- +1)
ITEM rec	Proportion HIT minus Proportion FA	Numeric (Range -1- +1)
ASSOC rec	Proportion HIT minus Proportion FA	Numeric (Range -1- +1)
NewVar7		
Response-latency-ITEM-BoL	Latency in msec. Item Primacy	Numeric (Range 0.00-9999.99)
Response-latency-ASSO-BoL	Latency in msec. Associative Primacy	Numeric (Range 0.00-9999.99)
Response-latency-ITEM-MoL	Latency in msec. Item Middle	Numeric (Range 0.00-9999.99)

Response-latency-ASSO-MoL	Latency in msec. Associative Middle	Numeric (Range 0.00-9999.99)
Response-latency-ITEM-EoL	Latency in msec. Item Recency	Numeric (Range 0.00-9999.99)
Response-latency-ASSO-EoL	Latency in msec. Associative Recency	Numeric (Range 0.00-9999.99)
total-latency	Latency in msec.	Numeric (Range 0.00-9999.99)
TOT-Latency-prim	Latency in msec.	Numeric (Range 0.00-9999.99)
TOT-Latency-middle	Latency in msec.	Numeric (Range 0.00-9999.99)
TOT-Latency-rec	Latency in msec.	Numeric (Range 0.00-9999.99)
TOT-latency-ITEM	Latency in msec.	Numeric (Range 0.00-9999.99)
TOT-latency-ASS	Latency in msec.	Numeric (Range 0.00-9999.99)
NewVar3		

- No missing values
